# The Syrian civil war: The experience of the Surgical Intensive Care Units

**DOI:** 10.12669/pjms.323.9529

**Published:** 2016

**Authors:** Hatice Kaya Ozdogan, Faruk Karateke, Mehmet Ozdogan, Sibel Cetinalp, Sefa Ozyazici, Yurdal Gezercan, Ali Ihsan Okten, Muge Celik, Salim Satar

**Affiliations:** 1 Hatice Kaya Ozdogan, MD. Dept. of Anaesthesiology and Reanimation, Adana Numune Training and Research Hospital, Adana, Turkey; 2 Faruk Karateke, MD. Dept. of General Surgery, Adana Numune Training and Research Hospital, Adana, Turkey; 3 Mehmet Ozdogan, MD. Professor of Surgery, Dept. of General Surgery, Medline Hospital, Adana, Turkey; 4 Sibel Cetinalp, MD. Dept. of Anaesthesiology and Reanimation, Adana Numune Training and Research Hospital, Adana, Turkey; 5 Sefa Ozyazici, MD. Dept. of General Surgery, Adana Numune Training and Research Hospital, Adana, Turkey; 6 Yurdal Gezercan, MD. Dept. of Neurosurgery, Adana Numune Training and Research Hospital, Adana, Turkey; 7 Ali Ihsan Okten, MD. Dept. of Neurosurgery, Adana Numune Training and Research Hospital, Adana, Turkey; 8 Muge Celik, MD. Dept. of Anaesthesiology and Reanimation, Adana Numune Training and Research Hospital, Adana, Turkey; 9 Salim Satar, MD. Associate Professor of Emergency Medicine, Dept. of Emergency Medicine, Adana Numune Training and Research Hospital, Adana, Turkey

**Keywords:** APACHE II, Civil war, Intensive care, Injury Severity Score (ISS), Syrian

## Abstract

**Objective:**

Since the civilian war in Syria began, thousands of seriously injured trauma patients from Syria were brought to Turkey for emergency operations and/or postoperative intensive care. The aim of this study was to present the demographics and clinical features of the wounded patients in Syrian civil war admitted to the surgical intensive care units in a tertiary care centre.

**Methods:**

The records of 80 trauma patients admitted to the Anaesthesia, General Surgery and Neurosurgery ICUs between June 1, 2012 and July 15, 2014 were included in the study. The data were reviewed regarding the demographics, time of presentation, place of reference, Acute Physiology and Chronic Health Evaluation II (APACHE II) score and Injury Severity Score (ISS), surgical procedures, complications, length of stay and mortality.

**Results:**

A total of 80 wounded patients (70 males and 10 females) with a mean age of 28.7 years were admitted to surgical ICUs. The most frequent cause of injury was gunshot injury. The mean time interval between the occurrence of injury and time of admission was 2.87 days. Mean ISS score on admission was 21, and mean APACHE II score was 15.7. APACHE II scores of non-survivors were significantly increased compared with those of survivors (P=0.001). No significant differences was found in the age, ISS, time interval before admission, length of stay in ICU, rate of surgery before or after admission.

**Conclusion:**

The most important factor affecting mortality in this particular trauma-ICU patient population from Syrian civil war was the physiological condition of patients on admission. Rapid transport and effective initial and on-road resuscitation are critical in decreasing the mortality rate in civil wars and military conflicts.

## INTRODUCTION

Since the civilian war in Syria began, millions of Syrian refugees have migrated to the neighboring countries. According to the official data from Turkish Government, by July 18, 2014, approximately one million 103 thousands of Syrian refugees were registered in and outside of the 22 camps in 11 provinces in Turkey.^1^ Excluding caesarean sections and spontaneous deliveries, 43.197 surgical operations were carried on Syrian refugee population by April 19, 2014.^2^ Besides, thousands of seriously injured trauma patients from Syria were brought to Turkey for emergency operations and/or postoperative intensive care.

Adana Numune Training and Research Hospital provides tertiary care to a large population and is located very close to the Syrian-Turkey border. Between June 1, 2012 and July 15, 2014, a total of 234.233 Syrian patients were admitted to our hospital, 2842 of them were hospitalized, and 1812 surgical procedures were performed. Surgical care and the operations mostly consisted of orthopaedic procedures as stated by another health care centre in Turkey.^3^

The aim of the present study was to present the demographics and clinical features of the wounded patients in Syrian civil war admitted to the surgical intensive care units (ICUs) in a tertiary care centre.

## METHODS

Among the 2842 Syrian patients hospitalized in our centre between June 1, 2012 and July 15, 2014, 80 trauma patients admitted to the Anaesthesia, General Surgery and Neurosurgery ICUs were included in the study. The institutional guidelines for selection criteria were followed for ICU admission. The patients who were cared for in Coronary, Internal Medicine, Paediatrics and Newborn ICUs due to medical problems were excluded. The patients admitted to Burn Unit, those with elective operations and those admitted due to other causes of trauma (e.g. traffic accidents) were also excluded. The hospital records of the patients were analyzed retrospectively. The data were reviewed regarding the demographics, time of presentation, place of reference, Acute Physiology and Chronic Health Evaluation II (APACHE II) score and Injury Severity Score (ISS), surgical procedures, complications, length of stay and mortality.

### Statistical Analysis

SPSS Statistics 21® software was used for the statistical analysis. Nominal variables were compared using the chi-square test. The differences in values between the survivors and non-survivors were assessed by Mann-Whitney U test. *P*<0.05 was considered as significant. Continuous data were presented as mean±SEM.

## RESULTS

During the study period, a total of 80 wounded patients (70 males and 10 females) with a mean age of 28.7 years were admitted to surgical ICUs. Anaesthesia ICU received 22 patients, General Surgical ICU received 39 patients, and 19 patients were admitted to Neurosurgical ICU. The patients were managed with a multi-disciplinary approach.

All injuries were combat-related. The most frequent cause of injury was gunshot injury (n=51, 63.75%). Nineteen patients (23.75%) had blast injuries, and the others had different injury patterns (Shrapnel – blast fragmentations and building collapses). The mean time interval between the occurrence of injury and time of admission was 2.87±0.48 days. Most of patients were referred from a primary or secondary health-care facility (n=59, 73.75%) and the others were referred directly to our hospital from field medical points in Syria. Thirty-five of the patients (43.75%) had had an operation before the referral. Forty-nine of the patients (61.25%) were operated on in our hospital after admission. There was no significant difference in APACHE II scores or mortality between patients who received surgery before referral to our centre, and those who were operated on after admission (*P*=0.297 for APACHE II, and *P*=0.308 for mortality).

The most common surgical procedures were orthopaedic surgeries (n=19) and second-look laparotomies including removal of packs (n=18). One anatomical site was injured in 36 patients (45%). Twenty-six patients (20.8%) had two different anatomical sites of injury. Nine, 7, and 2 patients had 3, 4, and 5 different sites of injury, respectively. Missed injuries, all of which were non-fatal and most of which were extremity-related, were detected in 24 (30%) patients.

Mean ISS score on admission was 21±1.25 and mean APACHE II score was 15.65±1.0. Mechanical ventilation was performed in 44 (55%) of the patients. The most common reason for mechanical ventilation was acute respiratory distress syndrome. Microbiological studies revealed bacterial overgrowths in blood (n=32, 40%), urinary (n=16, 20%), sputum (n=24, 30%), and wound (n=28, 35%) samples. Complication rate was 75% (n=60). The most common complication was pneumonia (n=39). Mortality rate was 55% (n=44).

**Fig.1:**
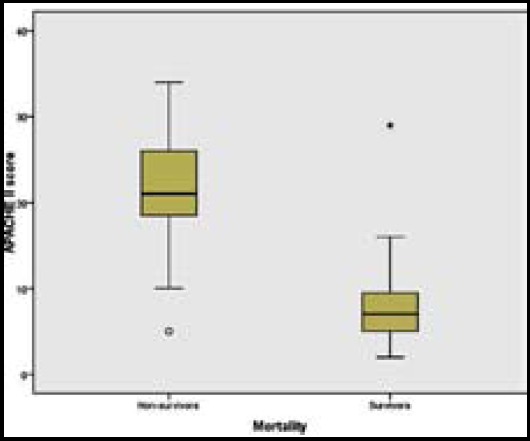
The difference in APACHE II scores in survivors and non-survivors.

Demographics and patient characteristics of survivors and non-survivors are presented in table 1. Mortality rate in males was significantly higher than females. APACHE II scores of non-survivors were significantly increased compared with those of survivors (Fig.1). A significantly prolonged length of mechanical ventilation was found in non-survivors. More complications occurred in non-survivors, as expected. When survivors and non-survivors were compared, no significant differences were found in the age, ISS, time interval before admission, length of stay in ICU, rate of surgery before or after admission, pattern of injury and number of injured anatomical sites. There was no significant difference in mortality in patients admitted to different ICUs (Table-II).

**Table-I:** Demographics and patients characteristic of survivors and non-survivors. Data are presented as mean±SEM.

	*Non-survivors*	*Survivors*	*p*
Age	27.53±1.89	28.45±2.0	NS
Sex (M/F)	35/9	35/1	0.0017
ISS	19.0±1.53	22.30±1.85	NS
APACHE II	21.65±0.95	8.40±1.40	0.001
Interval before admission (day)	3.0±0.58	2.81±0.74	NS
ICU LOS (day)	14.14±3.68	9.33±2.46	NS
Mechanical ventilation	33/44	11/36	0.0001
Days on mechanical ventilation	11.36±3.13	2.33±1.30	0.016
Surgery before admission	17/44	18/36	NS
Surgery after admission	24/44	25/36	NS
Complication	43/44	17/36	0.001

NS: Non-significant, ICU LOS: Intensive Care Unit length of stay.

**Table-II:** Distribution of patients among ICUs. There is no significant difference in mortality in patients admitted to different ICUs.

	*ICU*			*Total*

	*General Surgery*	*Anesthesia and Reanimation*	*Neurosurgery*	
Non-survivors	12	20	12	44
Survivors	10	19	7	36
Total	22	39	19	80

## DISCUSSION

The destruction of health system and deterioration of health care in Syria due to the ongoing civil war has been well documented.^4^ Majority of the hospitals, health centres and ambulances have been severely damaged or become non-functional due to lack of staff, equipment, and medicine.^4^-^6^ Seriously injured trauma patients have been treated either under these handicapped situations, or in field hospitals established by NGOs such as Doctors without Borders (MSF) and Syrian American Medical Society (SAMS).^7^,^8^ Most patients have undergone damage-control procedures, and then have been transported to the neighbouring countries, Turkey, Lebanon, Jordan, and Israel.^3^,^7^,^9^,^10^ Turkey is the northern neighbour of Syria, and the border is very close to the main conflict areas and Syrian cities such as Aleppo and Hama. That’s why many seriously injured patients has arrived in Turkey since the beginning of the war.^3^

The trauma mortality has a well-known trimodal distribution with almost half of deaths occurring instantaneously or during early pre-hospital phase. However, most casualties of low-intensity conflicts have been reported to die very early after injury, and it has been suggested that there is no trimodal death distribution in military trauma.^11^ The one of the most important determinants of survival is the rapid transport of casualties from the field of conflict to surgical facilities, and then to an intensive care environment. Most of our patients were referred to ICUs in our hospital from another health care facility. The mean time interval was almost three days, which is a very prolonged figure for a seriously traumatized patient. Despite nearly half the patients had been operated on before the admission, the prolonged transport under inappropriate conditions might have easily resulted in irreversible deterioration of patients’ physiological states and in a high mortality rate of 55% in our study population. Although, it could be assumed that the patients who received surgery prior to admission to ICU had lower APACHE II scores, and thus location of surgery might be a stronger predictor of outcome than APACHE II, this was not the case in our patient population. There were no significant differences in APACHE II scores and in mortality between patients who had received surgery before referral to our centre, and those who were operated on after admission. In the present study, APACHE II scores of non-survivors were significantly increased compared with those of survivors, reflecting the importance of the physiological state of the wounded patient. It can simply be assumed that principals of the damage control resuscitation,^12^ such as the prevention of hypothermia and haemostatic resuscitation, could not be applied to our patient population during transportation, at least until they reached the Syrian-Turkish border.^7^

During the war, the number of people wounded can be at least twice the number of killed and may increase 13 times as high.^13^ This ratio of the number of wounded to the number of killed results from the impact of the weapon systems in the particular context of war.

Injuries due to shrapnel have been reported to result in more mortality compared to the injuries by bullets.^14^-^17^ Most of our patients were injured by bullets, followed by shrapnel. Mortality rates are similar in different injury patterns in our study.

Depending on the context of the conflict, the injuries can show wide variation.^13^ A study from Croatia revealed that war injuries mostly involved muscle, soft tissue and bone, followed by abdomen, nervous system and thoracic region.^14^ A report from Israel, on the other hand, stated that the most seriously injured body parts were thorax, followed by head and abdomen.^11^ Two recent studies from Turkey showed that the most common diagnosis on admission was extremity injury^3^; however, head injury was the most common cause of death according to the autopsy reports of Syrian war casualties.^15^ More than half of the patients had multiple injured body parts.^15^ Our study also revealed that 55% of patients who required ICU admission had multiple injuries. However, the anatomical extent of the injury, namely ISS, was not found to have a significant effect on mortality in our study. Although civilian conflicts and bombings have been reported to have the highest mortality in the head-injured patients 15,16,18, no significant difference in mortality in patients admitted to the neurosurgical ICU was found compared to other trauma-ICU patients in our study.

### Limitations of the Study

Our civilian trauma population with a similar severity of illness, who were not transferred, has a lower mortality ratio. This is thought to be the result of unadjusted confounding, the consequence of the fact that deteriorating patients are more likely to be transferred than those who are improving. The other one is the lack of association of various possible mortality predictors might be the result of our insufficient sample size in the present study.

## CONCLUSION

In conclusion, the most important factor affecting mortality in this particular trauma-ICU patient population from Syrian civil war was the physiological condition of patients on admission. Rapid transport and effective initial and on-road resuscitation are critical in decreasing the mortality rate in civilian wars and conflicts.
